# Predictive Population Dynamics of Escherichia coli O157:H7 and Salmonella enterica on Plants: a Mechanistic Mathematical Model Based on Weather Parameters and Bacterial State

**DOI:** 10.1128/aem.00700-23

**Published:** 2023-06-22

**Authors:** Maria T. Brandl, Renata Ivanek, Ana Allende, Daniel S. Munther

**Affiliations:** a Produce Safety and Microbiology Research Unit, Agricultural Research Service, U.S. Department of Agriculture, Albany, California, USA; b Department of Population Medicine and Diagnostic Sciences, College of Veterinary Medicine, Cornell University, Ithaca, New York, USA; c Research Group of Microbiology and Quality of Fruit and Vegetables, Food Science and Technology Department, CEBAS-CSIC, Murcia, Spain; d Department of Mathematics and Statistics, Cleveland State University, Cleveland, Ohio, USA; The Pennsylvania State University

**Keywords:** STEC, Shiga toxins, abiotic factor, dormancy, field, human pathogen, leafy greens, modeling, phenotypic switch, prediction

## Abstract

Weather affects key aspects of bacterial behavior on plants but has not been extensively investigated as a tool to assess risk of crop contamination with human foodborne pathogens. A novel mechanistic model informed by weather factors and bacterial state was developed to predict population dynamics on leafy vegetables and tested against published data tracking Escherichia coli O157:H7 (EcO157) and Salmonella enterica populations on lettuce and cilantro plants. The model utilizes temperature, radiation, and dew point depression to characterize pathogen growth and decay rates. Additionally, the model incorporates the population level effect of bacterial physiological state dynamics in the phyllosphere in terms of the duration and frequency of specific weather parameters. The model accurately predicted EcO157 and S. enterica population sizes on lettuce and cilantro leaves in the laboratory under various conditions of temperature, relative humidity, light intensity, and cycles of leaf wetness and dryness. Importantly, the model successfully predicted EcO157 population dynamics on 4-week-old romaine lettuce plants under variable weather conditions in nearly all field trials. Prediction of initial EcO157 population decay rates after inoculation of 6-week-old romaine plants in the same field study was better than that of long-term survival. This suggests that future augmentation of the model should consider plant age and species morphology by including additional physical parameters. Our results highlight the potential of a comprehensive weather-based model in predicting contamination risk in the field. Such a modeling approach would additionally be valuable for timing field sampling in quality control to ensure the microbial safety of produce.

**IMPORTANCE** Fruits and vegetables are important sources of foodborne disease. Novel approaches to improve the microbial safety of produce are greatly lacking. Given that bacterial behavior on plant surfaces is highly dependent on weather factors, risk assessment informed by meteorological data may be an effective tool to integrate into strategies to prevent crop contamination. A mathematical model was developed to predict the population trends of pathogenic E. coli and S. enterica, two major causal agents of foodborne disease associated with produce, on leaves. Our model is based on weather parameters and rates of switching between the active (growing) and inactive (nongrowing) bacterial state resulting from prevailing environmental conditions on leaf surfaces. We demonstrate that the model has the ability to accurately predict dynamics of enteric pathogens on leaves and, notably, sizes of populations of pathogenic E. coli over time after inoculation onto the leaves of young lettuce plants in the field.

## INTRODUCTION

The nature of enteric-pathogen population dynamics that may explain pathogen survival on plants and result in foodborne illness has proven difficult to dissect at the macroscale of field studies. It is assumed that enteric pathogens die off due to a lack of fitness on plants and the risk of microbial contamination of the crop at harvest is hence diminished. Field studies have demonstrated that enteric-pathogen populations indeed decline rapidly over time after their inoculation onto the aerial parts of plants and that their survival frequently follows overall biphasic decay patterns (1–6). However, close examination of population data for enteric pathogens in plant inoculation studies reveals that survival trends also vary broadly.

Based on the analysis of field data compiled from different studies, McKellar et al. reported that the biphasic Weibull and Cerf’s models were better suited than a simple log-linear model to predict survival of Escherichia coli O157:H7 (EcO157) on lettuce plants in the field (7). Importantly, small pathogen populations that make up the tail of biphasic curves, and which may be more difficult to detect, can lead to underestimated risk (7, 8). Belias et al. also proposed a bisegmented log-linear model describing the first (rapid die-off) and second (slow die-off) phases of E. coli and Salmonella enterica population decay after their introduction onto leafy green plants in field plots in California, New York, and Spain (1). However, enteric-pathogen population growth rather than decay in the first segment was observed in 7.9% (11/140) of the plots in this study (1), reflecting substantial departure from a biphasic decay model.

Exposure of enteric bacteria to a range of stresses on plant surfaces has been the focus of causality of their common population decline in the phyllosphere under field conditions. There is strong evidence from investigations of epiphytic bacteria at the microscale that single-cell behavior within a population varies depending on the heterogeneous environment of the leaf surface and that multiplication events at discrete sites occur (9). Under laboratory conditions of warm temperature and the presence of free water on the leaves, enteric pathogens display a broad distribution of colony size and have the ability to increase in overall population size (10–12). Conditions of warm temperature and leaf wetness undoubtedly occur on plants in the field, even if only at distinct microsites and/or transiently, like during dew formation, rain, or irrigation, and provide opportunities for enteric pathogens to multiply in the absence of other inhibitory factors. Our mathematical model predicting the formation of persister variants of S. enterica on lettuce and spinach plants in the field revealed a negative correlation with weather factors promoting the presence of water on plants in the field (13). This is an additional indication that epiphytic enteric pathogens likely experience growth-conducive physicochemical events and not solely stress-mediated dormancy or death in the phyllosphere.

There is a critical lack of models that can predict survival of enteric pathogens and colonization of the phyllosphere taking into account the complexity and heterogeneity of their behavior and that of the physicochemical nature of plant surfaces. Failure to consider that enteric pathogens may multiply on plants limits our ability to predict when human health risk from consumption of plant-derived food may be highest. Pathogen multiplication on plants may occur as rare events at the subpopulation level that are overshadowed by net population decay or as rare events at the population level at large. Using available data from previously published lab studies on the population dynamics of enteric pathogens on plants, we developed here a mathematical model that integrates both enteric-pathogen population decline and growth to better predict the risk of contamination of edible crops. We further applied our model to EcO157 population dynamics on field-cultivated lettuce reported by Moyne et al. (5) in relation to the magnitude of three major environmental factors affecting bacteria on plant surfaces in the field, namely, water availability, temperature, and solar radiation intensity.

## RESULTS

### Model fitting and prediction of E. coli population dynamics in plant chamber studies.

**FIG 1:**
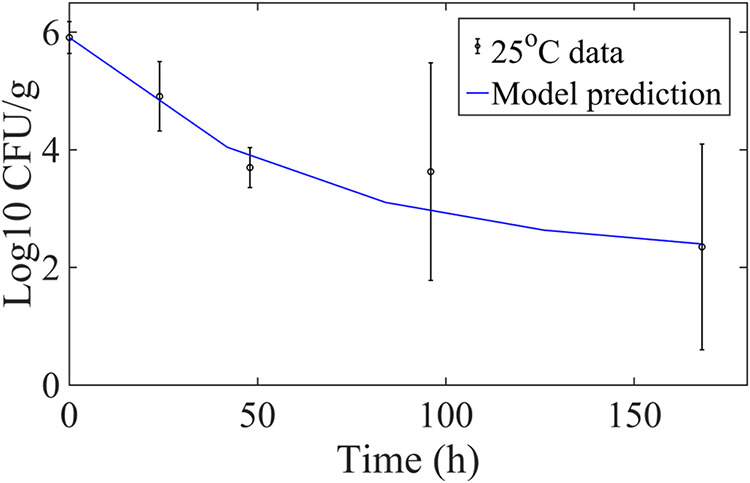
Model prediction of EcO157 population dynamics on lettuce leaves (blue line) under population decay conditions at 78% RH and 25°C using data from the study by Ottoson et al. (14) with an RMSE of 0.75 log_10_ CFU/g. Datum points represent measured mean population sizes and SD.

Table 1 provides an overview of the fitting and prediction results for this section. For E. coli, the study by Ottoson et al. (14) was utilized, in which survival studies of EcO157 on lettuce were conducted in climate chambers under controlled conditions of temperature, relative humidity (RH), and light intensity. Using EcO157 population data for 11°C and 18°C (RH fixed at 78% in both experiments) from Fig. 1 in reference 14, the fitting analysis revealed that an *a* value of 0.019/(h·°C) and a λ value of 0.016/h provided the best fit in terms of minimizing the modified function (equation 9) of the root mean square error (RMSE) with respect to both trials simultaneously. Fitting model equation 3 for *b*, using data from Fig. 3 in reference 14 with conditions of 18°C, 78% RH, and light intensity of 400 mmol/(m^2^·s), a *b* value of 70 mmol/(m^2^·s) provided the best fit, with an RMSE of 0.72 log_10_ CFU/g.

**TABLE 1:** Fit and prediction procedure for model application to EcO157 on lettuce leaves using the study by Ottoson et al. (14)

Step	Conditions	Parameter(s) fitted	Parameters used for prediction	Illustrated results
1. Fit	RH = 78%, temp = 11°C, 18°C	*a* = 0.019/h·°C, λ = 0.016/h		
2. Fit	RH = 78%, temp = 18°C, *L* = 400 mmol/m^2^·s	*b* = 70 mmol/m^2^·s		
3. Predict	RH = 78%, temp = 25°C, *L* = 400 mmol/m^2^·s		*a* = 0.019/h·°C, λ = 0.016/h, *b* = 70 mmol/(m^2^·s)	Fig. 1

**FIG 2:**
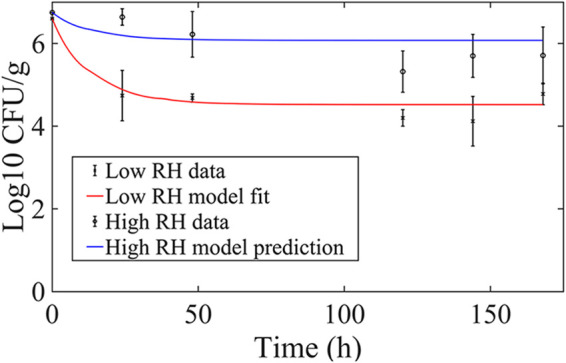
Model fit and prediction results for S. enterica populations on lettuce for fixed low- and high-RH conditions, fluctuating temperature (repeated 24-h cycles of 12 h of 23°C and then 12 h of 18°C) and fluctuating irradiance [repeated 24-h cycles of 12 h of 275 mmol/(m^2^·s) and then 12 h of 0 mmol/(m^2^·s)] from the study by Lopez-Galvez et al. (15). The red line illustrates the fit of model equations 3 to 7 to the S. enterica survival subject to the low-RH regime (RH = 60%) (RMSE = 0.56 log_10_ CFU/g compared to the 2-norm of the SD of data, which was 0.92 log_10_ CFU/g). The blue line represents the model prediction under the high-RH regime (RH = 85%) (RMSE = 1.0 log_10_ CFU/g compared to the 2-norm of the SD of the measured population, which was 1.2 log_10_ CFU/g). Datum points represent measured mean population sizes and SD.

Under decay conditions with light intensity dynamics, using a value for *a* of 0.019/(h·°C), a value for λ of 0.016/h, and a value for *b* of 70 mmol/(m^2^·s) in model equations 3 to 7 accurately predicted the E. coli population dynamics at 25°C (data from Fig. 2 in reference 14); importantly, the only change in the experimental conditions was a temperature increase from 18 to 25°C. Figure 1 illustrates the validation of both the model forms in equations 4 to 7 as well as the parameter values reflecting the experimental conditions set in that study. The prediction in Fig. 1 has an RMSE of 0.75 log_10_ CFU/g (comparing the average observed E. coli population numbers and the model prediction across all time points) and is well within the 2-norm of the standard deviation (SD) of the observed data (given the SD*_i_* at each time *t_i_*, the 2-norm is defined as ${\left[\underset{i}{\sum }{\left({\text{SD}}_{i}\right)}^{2}\right]}^{\frac{1}{2}}$), which was 2.7 log_10_ CFU/g.

### Model application to S. enterica population dynamics on baby lettuce in plant chamber studies.

Table 2 provides an overview regarding the following model fitting and prediction results of model equations 3 to 7 applied to S. enterica survival data on baby lettuce obtained in plant growth chambers by López-Gálvez et al. (15). Using a value for *b* of 70 mmol/(m^2^·s), the value established for E. coli population predictions, the model was fitted to data from the low-RH condition (RH = 60%) in Fig. 1A in reference 15, resulting in a value for *a* of 0.028/(h·°C) and a value for λ of 0.073/h. Considering only an increase in RH (60% to 85%), the population dynamics in Fig. 1A in reference 15 were predicted, as illustrated inFig. 2. In particular, the high-RH prediction had an RMSE of 1.0 log_10_ CFU/g compared to the 2-norm of SD of the measured population, which was 1.2 log_10_ CFU/g.

**TABLE 2:** Fit and prediction procedure for model application to S. enterica on baby lettuce leaves using the study by Lopez Galvez et al. (15)

Step	Conditions	Parameters fitted	Parameters used for prediction	Illustrated results
1. Fit	Fixed RH = 60%, variable temp and light intensity	*a* = 0.028/h·°C, λ = 0.073/h		Fig. 2
2. Predict	Fixed RH = 85%, variable temp and light intensity		*a* = 0.028/h·°C, λ = 0.073/h, *b* = 70 mmol/(m^2^·s)	Fig. 2
3. Fit	Dynamic RH (low), variable temp and light intensity	λ = 0.037/h, *a* = 0.028/h·°C (fixed from step 1)		Fig. 3A
4. Fit	Dynamic RH (high), variable temp and light intensity	λ = 0.023/h, γ = 0.0078/h, *a* = 0.028/h·°C (fixed from step 1)		Fig. 3B

**FIG 3:**
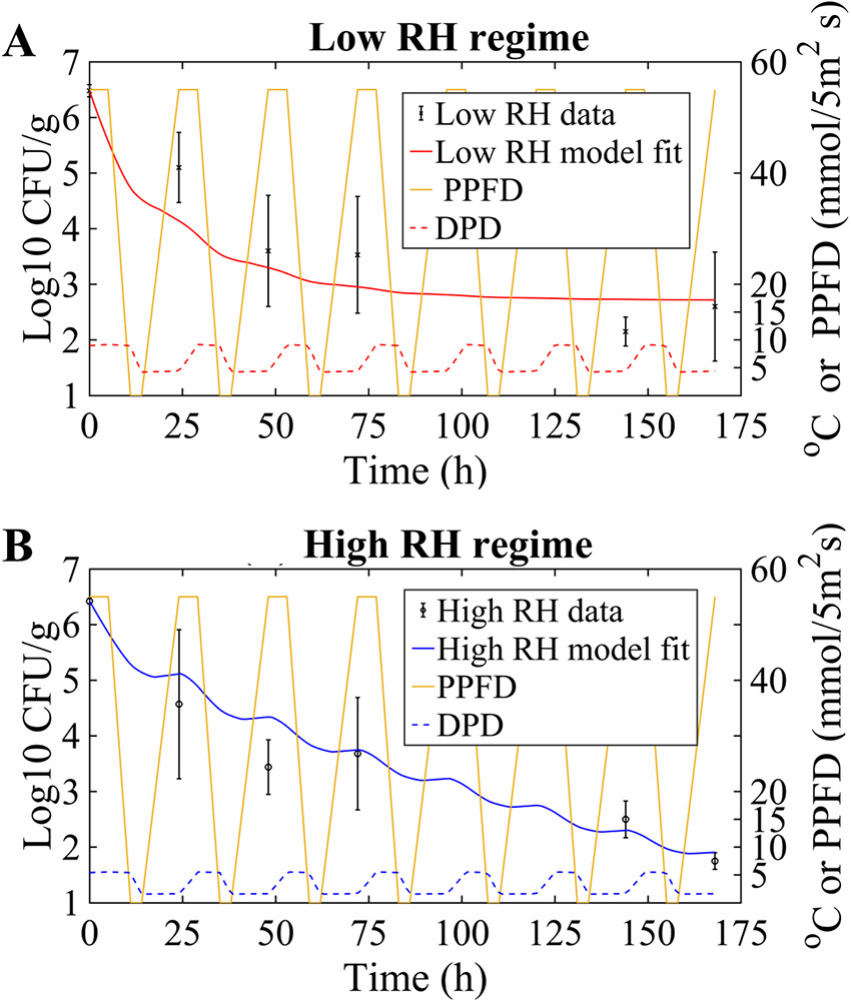
(A) Model fit (solid red line) to S. enterica population data on lettuce from the dynamic low-RH regime used by López-Gálvez et al. (15). The RMSE was 1.40 log_10_ CFU/g. The DPD (dotted red line) and irradiance (photosynthetic photon flux density [PPFD]) (yellow line) are shown. (B) Model fit (solid blue line) to S. enterica population data on lettuce from the dynamic high-RH regime used by López-Gálvez et al. (15). The RMSE was 1.08 log_10_ CFU/g. DPD (dotted blue line) and irradiance (PPFD) (yellow line) are shown. During periods when the DPD was less than the 2°C threshold value, the model predicts slight growth. Datum points represent measured mean population sizes and SD.

In addition to static RH conditions during their survival experiments, López-Gálvez et al. (15) examined S. enterica dynamics on baby lettuce under two sets of fluctuating RH conditions: high, ranging between 70 and 90%, and low, ranging between 55 and 75%. Given the temperature range for these experiments (10 to 18°C), the dew point depression (DPD; the difference between ambient temperature and dew point that is indicative of leaf wetness) for the low-RH regime is always above the 2°C threshold (Fig. 3A), and therefore, based on the model's structure, γ was not included in the parameter fitting. In contrast, γ was determined for the high-RH regime, during which the DPD periodically fell below 2°C (Fig. 3B), potentially providing free-water conditions on the leaves that may support bacterial growth.

Using a value for *a* of 0.028/(h·°C) from the static RH fit above, we found a value for λ of 0.037/h in connection with the dynamic low-RH regime (Fig. 3A), with an RMSE of 1.40 log_10_ CFU/g. Then, for the dynamic high-RH regime, we used a value for *a* of 0.028/(h·°C) and a value for μ*_g_* of 0.027/h (see the supplemental material for details regarding the growth rate). The resulting model fit is illustrated in Fig. 3B, where λ is 0.023/h and γ is 0.0078/h, with an RMSE of 1.08 log_10_ CFU/g.

Comparing the rate of switching from an active to a tolerant (inactive) state under the static low-RH regime (λ = 0.073/h) to λ values from the dynamic low-RH regime (λ = 0.037/h) and high-RH regime (λ = 0.023/h), a progressively lower switch rate is observed as RH values approach 100%. Hence, the DPD takes on values closer to and below the 2°C threshold, and the potential for free water on the plant surface increases, thereby improving the environmental quality and reducing the rate of switching to a tolerant inactive state. In addition, while the observed population data in Fig. 3A and B are statistically similar (comparing the population distributions with respect to each time point), our model indicates that these survival patterns occur for different reasons. In Fig. 3A, the model predicts only decay dynamics, which slow down as more cells switch to an inactive state. On the other hand, while the model also predicts an overall population decay trend in Fig. 3B, it specifically predicts successive decay/growth dynamics, with cells switching back and forth between inactive-tolerant and active states.

### Model application to S. enterica dynamics on cilantro in plant chamber studies.

We also applied our model in the context of S. enterica population dynamics on the leaves of cilantro plants, following data presented by Brandl and Mandrell (12) and Brandl (16). S. enterica cell populations were subjected to decay as well as extended growth periods on the leaves, which was controlled via low RH (50 to 60%) in a plant growth chamber and high RH (near 100%) in a dew chamber that promoted leaf wetness. While the cilantro plants in these experiments were subjected to light radiation postinoculation, the irradiation component of the decay form was not explicitly included in the model (therefore, *L* = 0 in model equation 6 for all model outputs in this subsection), since the light output was not recorded. In this case, the parameter *a* (equation 6) implicitly captures an average decay effect due to irradiation. Given the extended growth conditions in these experiments, the logistic growth form for μ*_g_* as given in equation 2 (see “Growth as a function of temperature” in Materials and Methods) was used. For an overview of the following fitting and prediction results, refer to Table 3.

**TABLE 3:** Fit and prediction procedure for model application to S. enterica on cilantro using data from the study by Brandl (16)

Step	Conditions	Parameters fitted	Parameters used for prediction	Illustrated results
1. Fit	Temp = 26°C, dry-wet	*a* = 0.02/h·°C, λ = 0.042/h, γ ∈ (0.0069, 0.013)/h		Fig. 4A
2. Predict	Temp = 26°C, wet-dry-wet		*a* = 0.02/h·°C, λ = 0.042/h, γ ∈ (0.0069, 0.013)/h	Fig. 4B
3. Fit	Temp = 26°C, wet-dry-wet	*a* = 0.02/h·°C, λ = 0.025/h, γ = 0.0017/h		Fig. 4B

**FIG 4:**
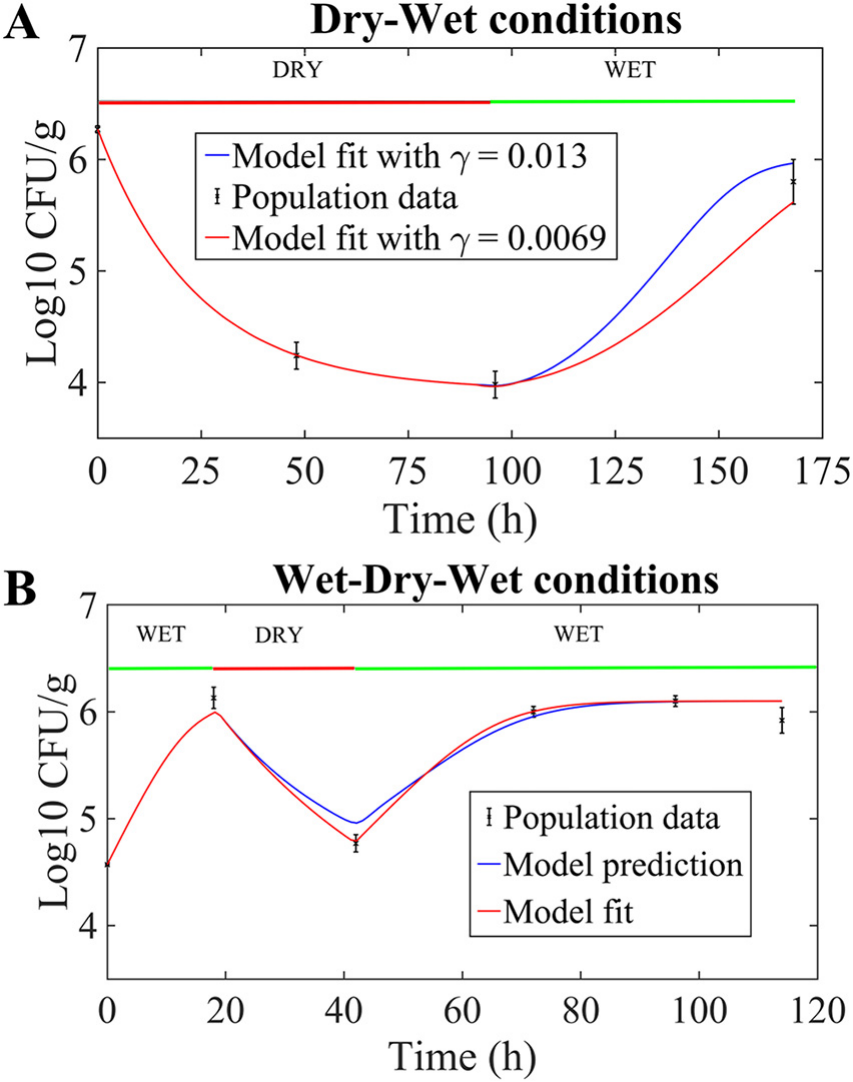
(A) Model fit results determining γ ∈ (0.0069, 0.013) for S. enterica data on cilantro under sustained dry-wet conditions in the study by Brandl (16). The range for γ was determined by constraining the model output to stay within standard error of the mean (SEM). (B) Model prediction (blue line) result for S. enterica data on cilantro under sustained wet-dry-wet conditions in the study by Brandl and Mandrell (12), where the best fit parameters (λ = 0.042; γ = 0.0089) from panel A were used. The RMSE was 0.26 log_10_ CFU/g. The best model fit (red line) (λ = 0.025; γ = 0.0017) for wet-dry-wet conditions, independent of the parameters in panel A. The RMSE was 0.23 log_10_ CFU/g. Datum points are illustrated as means and SEM.

For the “dry-wet” experiment described by Brandl (16), cilantro leaves were inoculated at approximately 6 log_10_ CFU/g, plants exposed to dry conditions (RH ≈ 50%, temperature = 26°C) for 96 h and then wet conditions (RH near 100%, temperature = 26°C) for the following 72 h. For the decay period (0 to 96 h), the best model fit determined that *a* was 0.02/(h·°C) and λ was 0.042/h (Fig. 4A). The growth dynamic, from 96 to 168 h postinoculation, was modeled using a value for μ*_g_* of 0.2621[1 − (*y*/*K*)], where 0.2621 quantifies the growth rate at 26°C and *K* is 6 log_10_ CFU/g (see the supplemental material for details). Using this growth form in model equations 3 to 7, the rate of switching back to the “active state” was γ ∈ (0.0069, 0.013), so that model outputs remained within the standard error bounds of the data at 168 h (Fig. 4A). In particular, the best fit from the wet part of the experiment was for a γ value of 0.0089/h, giving an RMSE of 0.01 log_10_ CFU/g.

The parameters *a* = 0.02/(h·°C), λ = 0.042/h, growth form μ*_g_* = 0.2621[1 − (*y*/10^6.1^)], and γ = 0.0089/h were then used to predict the experimental data in Fig. 6 in reference 12 under wet-dry-wet conditions. That is, inoculated cilantro was exposed to wet conditions (RH near 100%, temperature = 26°C) for 18 h, then dry conditions (RH near 60%, temperature = 26°C) from 18 to 42 h, and then wet conditions again (RH near 100%, temperature = 26°C) from 42 to 114 h. Our model prediction is illustrated in Fig. 4B, with an RMSE of 0.26 log_10_ CFU/g. The model accurately captures the sequential growth-decay-growth dynamics, and most of the error in the prediction comes from the dry (decay) regime. Indeed, the best model fit for the wet-dry-wet experiment gave a value for λ of 0.025/h and for γ of 0.0017/h, with an RMSE of 0.23 log_10_ CFU/g. Note that the wet-dry-wet best-fit λ is about 1.7 times smaller than the dry-wet λ, indicating that the switch to tolerance is slower in the wet-dry-wet experiment. That is, λ may be a function of the history of the intensity and length of the stress period experienced by the bacterial cells. In terms of net population dynamics, the red curve (model fit) in Fig. 4B illustrates that this lower switch rate translates into a slightly higher population decay rate (from 20 to 40 h) compared with the blue curve predicted from the dry-wet conditions.

### Model application to EcO157 dynamics on lettuce in a California field study.

Model equations 3 to 7 were applied to the field study by Moyne et al. (5), which investigated EcO157 dynamics after inoculation onto romaine lettuce in the field in Salinas, CA. As the model requires detailed weather inputs (i.e., solar radiation intensity, temperature, and RH), data collected during 15-min intervals from CIMIS weather station 89 (https://cimis.water.ca.gov) located in close proximity to the field trials in the latter study were used. Following the experimental design in this study, the analysis was separated into two categories depending on the time of inoculation, morning (7:30 to 9 a.m.) versus night (9 to 11 p.m.). Note that we did not include trial 5, 6-week-old plants (morning and night inoculations) in our analysis because detailed weather data were not available for these trials in the study. For a summary of the following fitting and prediction results, see Table 4.

**TABLE 4:** Fit and prediction procedure for model application to EcO157 on romaine lettuce leaves using the field study by Moyne et al. (5)

Step	Conditions^*a*^	Parameters fitted	Parameters used for prediction	Illustrated results
1. Fit	Morning inoculation trial, (T3 6wk M)	*a* = 0.11/h·°C, λ = 0.11/h		Fig. 5A
2. Predict	Other morning inoculation trials		*a* = 0.11/h·°C, λ = 0.11/h, *b* = 100 W/m^2^, γ ≈ 0.001 or 0.01/h	Fig. 6
3. Fit	Night inoculation trial (T3 6wk N)	*a* = 0.11/h·°C (fixed from step 1), λ = 0.08/h		Fig. 5B
4. Predict	Other night inoculation trials		*a* = 0.11/h·°C, λ = 0.08/h, *b* = 100 W/m^2^, γ ≈ 0.001 or 0.01/h	Fig. 7

a T3 6wk M, trial 3, 6-week-old plants with morning inoculation; T3 6wk N, trial 3, 6-week-old plants with night inoculation.

### (i) Model fitting and predictions for morning inoculation trials.

**FIG 5:**
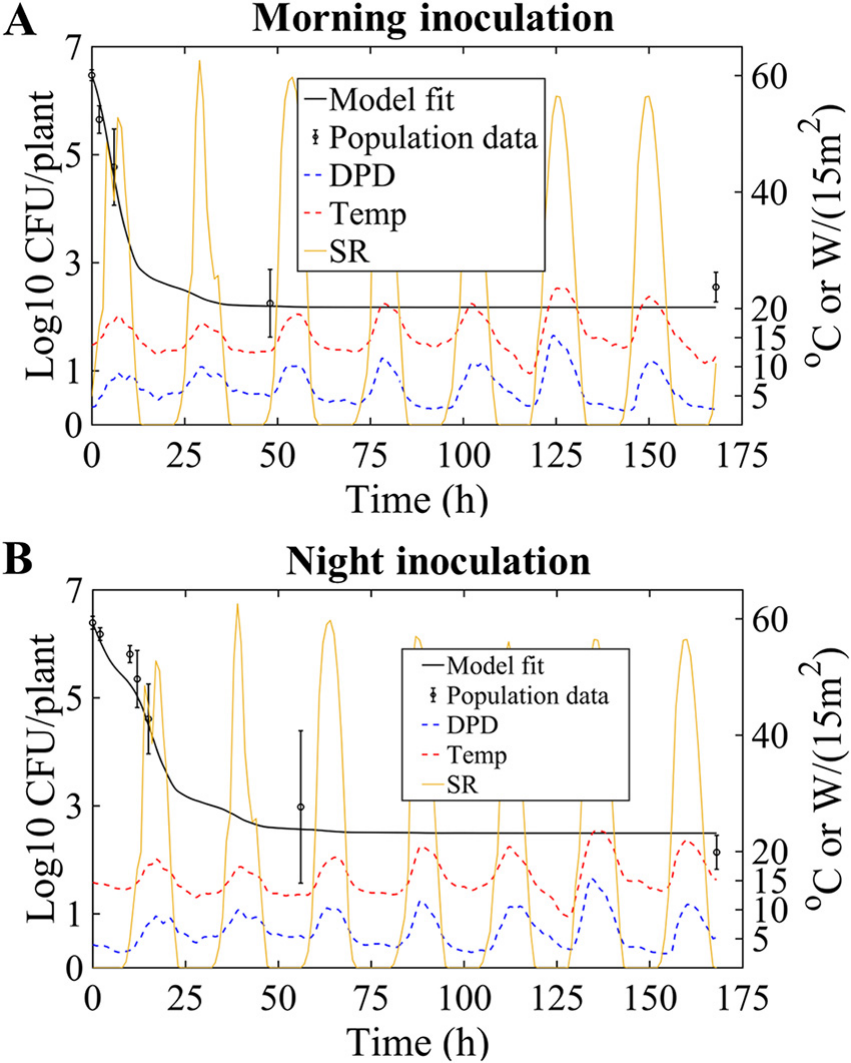
Model fitting results for EcO157 dynamics after morning or night inoculation of 6-week-old romaine lettuce in trial 3 in the field study by Moyne et al. (5). Population data are presented as means with 95% confidence intervals (CI) (error bars). Yellow curves depict solar radiation (SR), dotted red curves illustrate temperature (Temp), and blue dotted curves represent DPD. (A) Model fit (solid black curve) to population data from morning inoculation, determining *a* as 0.11/(h·°C) and λ as 0.11/h with an RMSE of 0.55 log_10_ CFU/plant. (B) Model fit (solid black curve) to population data from night inoculation using an *a* value of 0.11/(h·°C) to determine a λ value of 0.08/h, with an RMSE of 0.90 log_10_ CFU/plant.

Regarding trials with morning inoculation times, the model was fitted to population data from trial 3, 6-week-old plants (morning inoculation) to determine a value for *a* of 0.11/(h·°C) and for λ of 0.11/h, with a global (across all time points) RMSE of 0.55 log_10_ CFU/plant (Fig. 5A). This trial was selected since only “decay” parameters were necessary to fit, as the DPD never drops below 2°C.

Using the parameter information *a* = 0.11/(h·°C), λ = 0.11/h, and *b* = 100 W/m^2^ (see the supplemental material for more details regarding the value of *b* in this case) as model inputs, the equation 1 growth form μ*_g_* = (1.265 × 10^−4^) × [*T*(*t*) − 2.85]^2^, informed by data from the work of Ratkowsky et al. (17) and Simko et al. (18), the respective weather data, and the initial population, model equations 3 to 7 were utilized to predict E. coli dynamics from the other morning inoculation trials. During predicted periods of growth (that is, when the DPD was ≤2°C), we used two values of γ (the rate at which bacteria resume growth), ~0.001/h and ~0.01/h, to illustrate a range of model predictions. Given the lack of detailed time series data during potential growth periods in the field trials by Moyne et al. (5), the model fits for γ cannot be uniquely determined. For the best approximation, these values were chosen from a similar range determined as described above from the model application to S. enterica on lettuce and cilantro.

**FIG 6:**
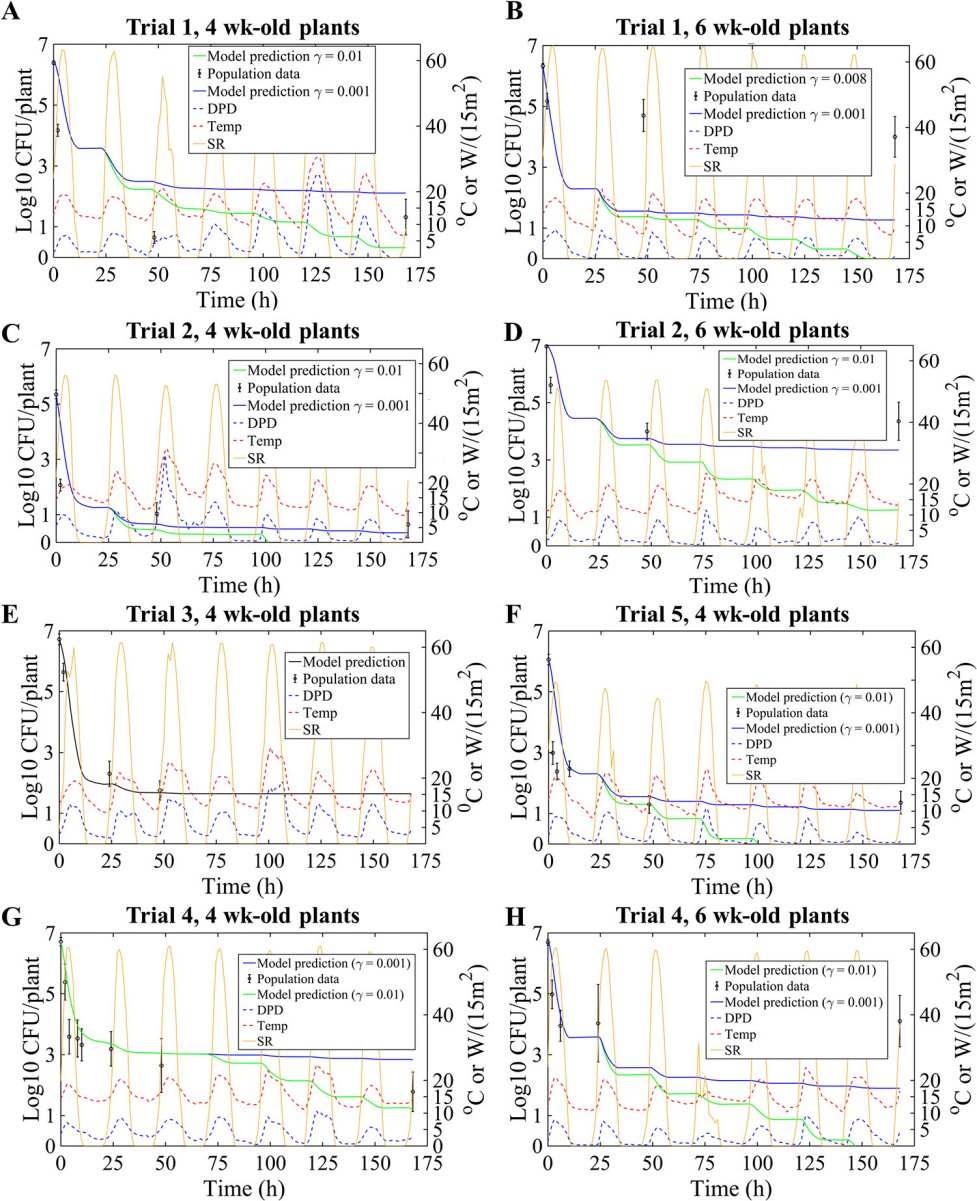
Model prediction results for EcO157:H7 dynamics after morning inoculation of 4-week- and 6-week-old romaine lettuce in various trials in the field study by Moyne et al. (5). Population data are presented as means with 95% CI (error bars). Yellow curves depict solar radiation (SR), dotted red curves illustrate temperature (Temp), and blue dotted curves represent DPD. RMSE ranges (log_10_ CFU per plant) are presented in connection with 0.001 ≤ γ ≤ 0.01 with respect to relevant trials. (A) Trial 1, 4-week-old plants. RMSE = (2.6, 2.7). (B) Trial 1, 6-week-old plants. RMSE = (4.2, 5.2). (C) Trial 2, 4-week-old plants. RMSE = (2.3, 3.1). (D) Trial 2, 6-week-old plants. RMSE = (1.6, 2.4). (E) Trial 3, 4-week-old plants. RMSE = 0.64 (no growth periods; therefore, γ is not used). (F) Trial 5, 4-week-old plants. RMSE = (3.0, 4.0). (G) Trial 4, 4-week-old plants. RMSE = (1.8, 2.0). (H) Trial 4, 6-week-old plants. RMSE = (2.5, 4.7).

Figure 6 illustrates the prediction results for morning inoculation trials. Model forms for bacterial growth and decay, equations 4 to 7, constructed and validated from information at the lab scale, had predictive merit at the field level. For the morning inoculation, the model correctly predicts the initial steep decay of the E. coli population 10 to 24 h after inoculation in terms of the respective solar radiation and DPD dynamics (Fig. 6). Importantly, the rate of switching to tolerance λ (per hour) (model equations 6 and 7) plays a key role in setting the average time scale for retarding the initial fast decay of the population. The fit value for λ of 0.11/h (Fig. 5A) and the predictive success of the model in Fig. 6 indicate that this time scale is consistently on the order of 10 h.

In addition to the initial decay, the model also accurately predicted the tail populations at ca. 24 to 175 h postinoculation in most of the morning inoculation trials (Fig. 6). This is significant given the length of time postinoculation and the dynamic weather conditions contrasting successive day/night periods. For most of these morning inoculation trials in Fig. 6, the DPD dropped below 2°C at night, and the model predicted slight growth. However, due to a low temperature of 10 to 15°C at night, the predicted growth rate was low (on the order of 6.5 × 10^−3^/h). In addition, model predictions using the smaller value of γ of 0.001/h (the rate of switching of cells from tolerance to a potential growing state) had less error, as the smaller γ value resulted in higher population sizes in the predicted tails. While the model anticipates that a larger γ value leads to more cells switching to an active state (when the DPD is <2°C), it also indicates that the susceptibility of the population increases. That is, as the population is again exposed to harsh conditions (i.e., solar radiation and high DPD), the predicted decay rate would be higher for the population that has more “active” cells. Since these low rates of growth and switching from tolerance resulted in overall steady population dynamics (nighttime) instead of population size increases, coupled with a lower decay rate (daytime), the model predicts larger tail populations with smaller γ values. This is illustrated by the blue curves in Fig. 6, our model output that typically best describes the data, suggesting that the time scale to switch out of tolerance may have been quite long, on the order of 1,000 h. Additionally, our model predictions were generally better for 4- than 6-week-old plants in the trials by Moyne et al. (5), in particular those depicted in Fig. 6B and H. Overall, the smaller γ value provided a better fit in the case of older plants; however, both low and high γ values considerably underestimated the actual population sizes in the tail part of the dynamics when E. coli survived at an unexpectedly high rate (Fig. 6B and H).

### (ii) Model fitting and predictions for night inoculation trials.

**FIG 7:**
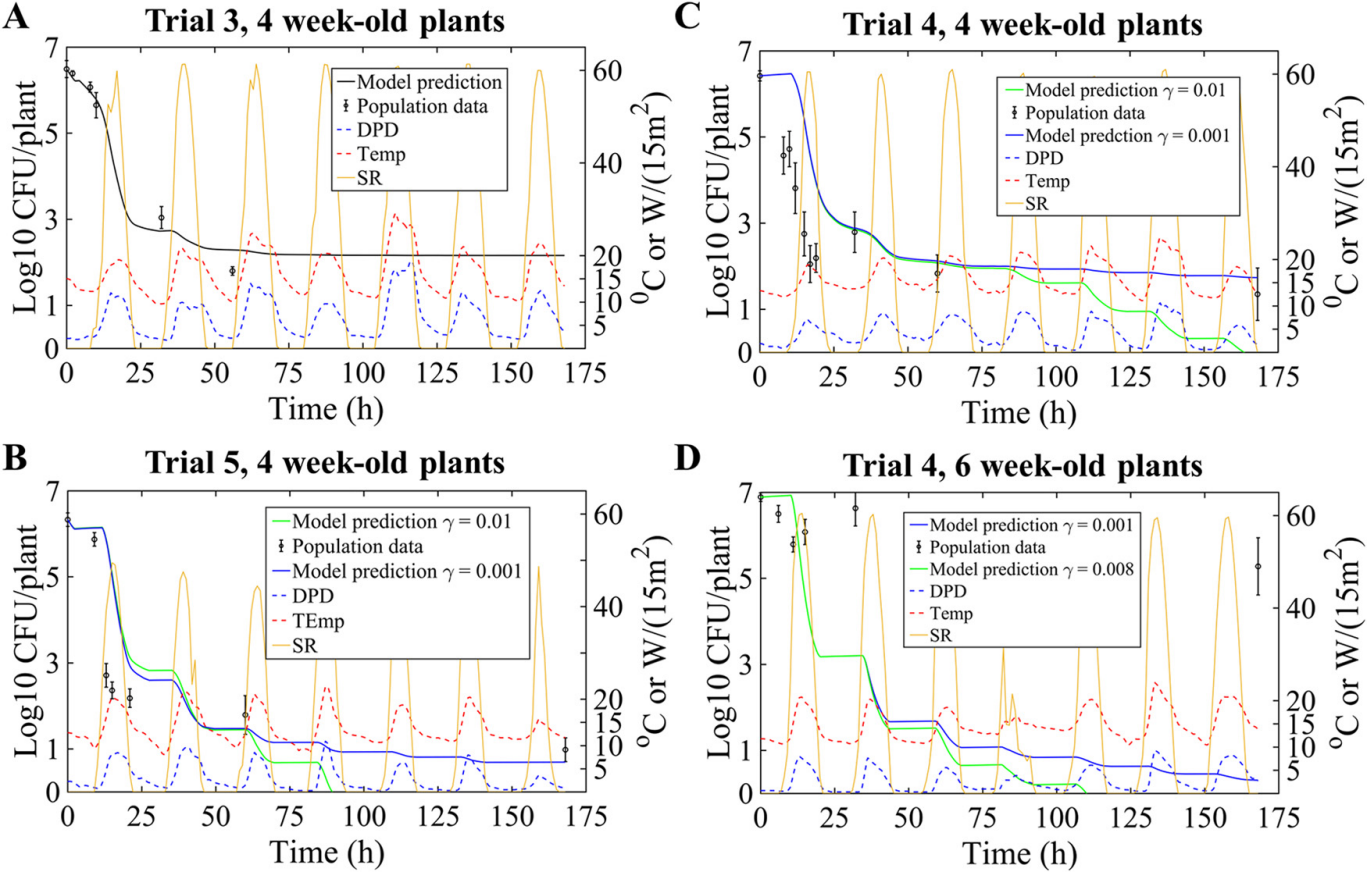
Model prediction results for EcO157:H7 dynamics after night inoculation of 4- and 6-week-old romaine lettuce various trials in the field study by Moyne et al. (5). Population data are presented as means with 95% CI (error bars). Yellow curves depict solar radiation (SR), dotted red curves illustrate temperature (Temp), and blue dotted curves represent DPD. RMSE ranges (log_10_ CFU per plant) are presented in connection with 0.001 ≤ γ ≤ 0.01 with respect to relevant trials. (A) Trial 3, 4-week-old plants. RMSE = 0.61 (no growth periods; therefore, γ is not used). (B) Trial 5, 4-week-old plants. RMSE = (4.2, 5.1). (C) Trial 4, 4-week-old plants. RMSE = (5.4, 5.6). (D) Trial 4, 6-week-old plants. RMSE = (8.19, 8.2).

In terms of determining model parameter inputs, a value of *a* of 0.11/(h·°C) was used from the morning inoculation trials, but λ, the rate of switching to tolerance, was fitted in this new context, as the switch rate may depend on the harshness of conditions at inoculation time. In particular, trial 3, 6-week-old plants (night inoculation) were used, since the DPD was not sufficiently low to permit growth conditions, resulting in a value for λ of 0.08/h with an RMSE of 0.90 log_10_ CFU/plant. The model best fit is illustrated in Fig. 5B. The model predictions for night inoculation, as presented in Fig. 7, show that in 3 of 4 trials, the model correctly predicted slow decay or slight growth following night inoculation. This gives credence to the DPD threshold encoded in model equations 3 to 7 for distinguishing overall decay versus growth periods. In contrast, irrespective of the γ value, the model failed to accurately predict long-term EcO157 population dynamics on 6-week-old lettuce plants, during which survival of the pathogen was very high after inoculation (Fig. 7D) compared to that on 4-week-old plants (Fig. 7A to C).

## DISCUSSION

Population decline is the most common trend observed after E. coli and S. enterica are inoculated onto plants in field studies. Consequently, mathematical models of enteric-pathogen survival on crops that have been developed in the context of food safety are limited to exhibiting mono- and biphasic-type decay dynamics. Recent studies have primarily utilized empirical models to describe frequently observed biphasic bacterial population decay patterns on plants (1, 7, 19–22). These decay models cannot predict bacterial trends across various abiotic conditions at the fine time scale, such as during growth-promoting conditions. In this study, we developed an original mathematical model that incorporates the following aspects: (i) weather information approximating an “average” local environment of either stressful or growth-promoting conditions for bacteria, with the nuanced consideration that both phenomena may occur concomitantly at separate microsites on the leaves but are not captured at the macroscale of our models; (ii) bacterial state dynamics (inactive-tolerant versus active); and (iii) mathematical forms that reflect observed scientific principles, consisting of biologically meaningful parameters. Our current knowledge of behavior of enteric pathogens on plants can be applied to comprehensive mechanistic models that explain pathogen growth and die-off patterns in terms of these aspects. For example, E. coli and S. enterica die-off patterns on preharvest lettuce and spinach leaves have been statistically associated with ambient relative humidity and dew point, and periods of growth have been observed (1). Furthermore, the physiological state of enteric pathogens at the subpopulation level is associated at least partly with abiotic factors such as leaf wetness, RH, and solar radiation (13, 23).

Bacterial exposure to an array of fluctuating physicochemical stresses has long been recognized as a major factor in their survival on plants (24). The high adaptability of enteric pathogens to numerous stresses (25–27) likely enables them to survive in secondary habitats and to exploit conditions conducive to their growth, even if only in spatiotemporally limited occurrences, such as are commonly encountered on plants. Our modeling study aimed directly at integrating such survival and growth phases to predict colonization of S. enterica and E. coli in the phyllosphere under fluctuating conditions of radiation, temperature, and free-water availability. When combined with growth-permissive temperature and the presence of nutrients, and in the absence of inhibitory factors, free water at microsites may enhance bacterial survival and promote multiplication by also increasing solubilization of substrates. Rain and crop irrigation represent major crop-wetting events, but periodic leaf surface wetness may also be caused by fog deposition, dew formation, and the microscopic water films and droplets resulting from the deliquescence of hygroscopic particles (28). The weather station near the field trials used in our study did not record any precipitation during the sampling period, and leaf wetness caused by fog deposition or deliquescence in field trials could not be considered in this work due to a lack of specific data. However, dew point could be computed from recorded temperature and RH data. Therefore, DPD, defined as the difference between ambient temperature and dew point that is indicative of leaf wetness (29), represented a major variable in our predictive model.

Microbes in the phyllosphere, including E. coli, are also greatly impacted by solar radiation (2, 3, 13, 30, 31). Hence, exposure to radiation was included along with DPD and temperature to assess the predictive value of our model. Data from the field study by Moyne et al. (5) used in this work were therefore particularly suitable to test our model in this regard, since EcO157 was reported to have greater survival after inoculation onto lettuce at night than in the morning.

Bacterial state is a critical consideration to effectively model population dynamics, especially in variable environments such as the phyllosphere. Clonal subpopulations of active and inactive bacteria are present on plant surfaces (9, 23, 32–35). To describe observed phenomena such as variable decay rates, injury, lag periods, and regrowth, subpopulation models classifying various bacterial states have been developed (23, 36–38). We assumed in our model that the bacterial population on a produce leaf is composed of two subpopulations, the sizes of which vary depending on abiotic conditions: active (able to grow) and inactive but more tolerant to stress. Therefore, our model tracks the total population on the leaf, incorporating a scaling function (*F*) to approximate the effects of subpopulation dynamics on the total population under stressful conditions when certain cells switch to a tolerant state and under growth conditions when certain cells switch back to an active state. The forms for *F* indicate the assumption that times between switching states are exponentially distributed. The distributions regulating switching times between active and inactive states have not been studied for bacteria in the phyllosphere, but recent studies have demonstrated lag time distributions with large variance during E. coli growth resumption following exposure to stressful environments (39, 40). Characteristics of switch time distributions, e.g., mean and variance, also may be functions of the ambient environment, and therefore, it is important to quantifiably link them to the environmental dynamics in the field. In a study investigating the effect of osmotic stress on the lag phase of S. enterica in culture, the initial population decay and lag time for regrowth increased with increasing salt concentration (41). In the context of model equations 3 to 7, these observations suggest that λ (per hour) and 1/γ (hours) may be increasing functions of environmental stress. Our results point to this trend for λ, both for S. enterica on cilantro in growth chambers and for EcO157 on lettuce in the field, but it is not clear if this relationship holds for 1/γ, since we cannot ensure the practical identifiability of this parameter with the current data.

Utilizing mathematical forms relative to conditions dictating both decay and growth, and with minimal parameters under a variety of conditions and for various bacterium/produce pairs, our model showed predictive success at both the laboratory and field scales. In the laboratory studies, the model captured observed phenomena such as consistent decay rates during stressful conditions of light intensity and high DPD, growth rates as a function of temperature, and the transition between decay and growth periods as set by the DPD threshold of 2°C. Most importantly, our model successfully predicted EcO157 population dynamics after inoculation onto romaine lettuce for nearly all trials in the field study for which weather parameters recorded by a weather station were available to test the model under abiotic conditions predictive of population decay or growth (Fig. 6 and 7). Thus, a highlight of our model is that it provides strong evidence for the predictive capability of solar radiation, dew point, temperature, and bacterial physiological state to forecast EcO157 population dynamics on preharvest lettuce. Our model is unique in that it links observed bacterial dynamics on leaf surfaces with the characteristic time scales of these predictors. For instance, it accurately captured the early phase of population decay, relative to solar radiation, large DPD, and switching to an inactive tolerant state in most trials with morning inoculation (Fig. 6). Furthermore, it approximated well the observed trend of significantly reduced early decay rates after night inoculation, i.e., in the absence of solar radiation and with low DPD (Fig. 7B and C), compared with morning inoculation. In this case, a DPD threshold value slightly lower than the value of 2°C used here, which was obtained for cotton leaves (29), would have shifted predicted decay curves within 24 h postinoculation leftward and thereby shortened the predicted growth period to match the measured population sizes more closely. One would expect that such parameter values acquired specifically for lettuce plants under field conditions would enhance predictive accuracy. Nevertheless, it is noteworthy that our model predicted the population decay rate very accurately (Fig. 7B and C), suggesting that the magnitude and timing of solar radiation and DPD information are correctly utilized in model equation 6 and 7.

In line with most reports about enteric-pathogen behavior after inoculation onto plants in field studies, our model did not predict EcO157 multiplication events as overall population size increases. Weather conditions with a DPD of <2°C and low solar radiation occurred in the early morning when temperatures were 10 to 15°C in the field trials by Moyne et al. (5). Consequently, our model predicted very low growth rates (0.0022 to 0.019/h) and a long time to switch out of tolerance (on the order of 1,000 h) during these time periods. The resulting dynamics under the latter conditions were those of overall steady pathogen populations, likely reflecting a contribution of these very low rates to overall population survival in a population in which other cells undergo death in the heterogeneous environment of the phyllosphere at the microscale. Note that our model could be applied to microscale data detailing population changes as a function of conditions (free water, nutrients, temperature, etc.) at specific microsites on the leaf. However, to consider, for instance, the dispersal effect of free water on the distribution of cells on the leaf in conjunction with spatially varying microsite conditions, our model would need to be enhanced to account for specific spatial and temporal factors (42). Such a model may provide important insight into how population changes across microsites scale up to overall population dynamics on the leaf (43, 44).

Model predictions of long-term survival of EcO157 on lettuce in the field were better for smaller values of γ (per hour) (the rate of switching from the inactive to active state during conditions promoting potential growth) (Fig. 6 and 7). This trend was independent of inoculation time (morning versus night) and indicates that, as expected during the typical harsh conditions on leaves in the field, EcO157 cells that are inactive on average require considerable time to revert back to a growth state. Whereas resuscitation of dormant enteric-pathogen cells from stress conditions in food has received attention for the purpose of accurate detection on culture media (45), little is known about the physiological shift of bacterial colonists out of stress and into proliferation in the phyllosphere. A better understanding of this transition in enteric pathogens on plants may inform risk assessment with regard to expected lag times during potentially growth-promoting weather events, especially close to harvest time. Additional population measurements during the period of transition from decay to growth would be required to more precisely determine γ. Note that our DPD threshold provides a guideline as to when such measurements are likely to be useful; i.e., samples should be collected when the DPD is <2°C. Although the average time to remain inactive (1/γ) is much larger than the nighttime duration in the field, given the underlying stochasticity of this time scale, there is a possibility that a small fraction of the cell population will switch to growth and hence pose a measure of risk, especially if the other conditions are coupled with warm temperatures. Given the low infectious dose of EcO157 (46), even a few cell generations during growth-permissive phases on the leaves may increase the risk to human health.

Whereas our model accurately predicted EcO157 population sizes on 4-week-old plants, it underpredicted those on 6-week-old plants over periods of long-term survival in some trials. This is possibly due to the denser canopy and more complex leaf topography of older romaine lettuce plants, which may offer bacteria greater protection from solar radiation and harbor water for extended periods of time even once the DPD increased above 2°C. Leaf wetness duration (LWD) is influenced by wind speed and radiation and is an important factor in microbial infection of plants (47). Although it also impacts epiphytic bacteria, it was not considered in this study due to a lack of available field data. Bacterial growth periods that occurred more frequently than the DPD threshold predicted because of extended LWD may therefore partly explain our observed discrepancies in certain predictions for 6-week-old plants. Hence, model equations 3 to 7 are appropriate for predicting the fate of enteric pathogens on the leaves of young plants but must be augmented for older plants, possibly by including LWD as an abiotic variable.

Overall, this new model aims at closing gaps in our ability to assess the risk of crop contamination by taking into account weather conditions and their effects on foodborne pathogens in the phyllosphere. Whereas modeling microbial behavior on plants based on weather inputs has been a powerful predictive tool in the management of plant disease, weather variables have received little attention so far in studies on produce safety. The microbiome of the lettuce phyllosphere and of soil adjacent to it is known to have seasonality (31, 48, 49). Additionally, EcO157 dynamics on cold-stored cut lettuce and lettuce shelf life vary depending on harvest season (50, 51). Outbreaks of disease caused by Shiga toxin-producing E. coli (STEC) linked to lettuce in the United States are known to show seasonality (52, 53), and season was identified as a risk factor in *Enterobacteriaceae* and E. coli densities on spinach and arugula cultivated in Sweden (54). Notwithstanding that seasonality of foodborne disease from crops likely results from numerous biotic and abiotic factors and their interaction (55), a focus on the impact of weather on the population dynamics of enteric pathogens on plants, as well as on the physicochemical properties of their plant host, seems imperative in current efforts to enhance produce safety. Our study attempted to synthesize the temporal interplay of weather factors and bacterial physiological state to inform risk. Further understanding in this regard and of bacterial behavior and abiotic conditions at a finer spatial scale will provide much-needed knowledge to empower risk assessment with predictive mathematical modeling tools.

## MATERIALS AND METHODS

### General approach to the model.

Let *y* be the total population size (in CFU per 100 g, CFU per gram, CFU, etc.) of the bacteria in question on some produce type. We employ the model form *dy*/*dt* = (μ − *k*)*y*, where the growth rate (μ) is $f\left[t,y,\vec{E}\left(t\right),\vec{F}(t)\right]$ and the decay rate (*k*) is $g\left[t,\vec{E}\left(t\right),\;\vec{F}(t)\right]$, where $\vec{E}(t)$ is some function of the ambient environment experienced by the bacteria and $\vec{F}(t)$ reflects information related to the state of bacteria in the population (either growing or nongrowing). The main thrust of this study was to build mechanistic forms for μ and *k* based on experimentally observed principles that have predictive capacity at both the lab and field scales. The following sections detail these forms in terms of environmental and biological factors affecting bacterial population dynamics.

### Model development. (i) Incorporation of bacterial state.

We suppose that the bacterial population is composed of two subpopulations on the leaf (9, 23): a subpopulation that is active (growing), denoted by *n*(*t*), and a subpopulation that is inactive (nongrowing) but has a certain tolerance to the leaf environment, designated *p*(*t*). An active cell is defined herein as one that is multiplying, whereas an inactive tolerant cell is one that is not multiplying but is capable of withstanding the stresses that underly that inactive state. Hence, the total population size at any time *t* is given by *y*(*t*) = *n*(*t*) + *p*(*t*). Note that our model does not integrate population sizes of active and inactive bacteria at the single bacterial scale, since this information is not available. Rather, our model assumes that net bacterial decay or growth occurs based on ambient abiotic parameters. However, any total bacterial population size at any time may result from cell death, growth, steady state, or any combination thereof in that population of cells.

During a period of net population decay, the population dynamics are represented by the following basic system: *n* = −*kn* − λ*n* and *p* = λ*n*, with *n*(0) = *y*_0_, *p*(0) ≈ 0, and *k* ≫ λ. Here, 1/*k* (hours) represents the expected lifetime of the active population and λ (per hour) is the rate at which cells become inactive with tolerance. By solving the above system for *n*(*t*) and *p*(*t*) explicitly, one can see that by time 1/λ (hours), the population decay has slowed considerably as the total population *n*(*t*) + *p*(*t*) approaches the positive steady state, *y*_0_λ/(*k* + λ). Based on this behavior, we can approximate the population dynamics during a decay regime only in terms of the total population *y*, written as *y* = −*ke*^−λ^*^t^y*. The benefit here is that subpopulation data are not necessary to inform model parameters, specifically λ, the rate at which the cells develop inactive tolerance. The term *e*^−λ^*^t^* is a fractional scaling of the decay rate of the total population, which sets the expected value of the effective decay period to be 1/λ (hours). Applying this notion during a decay period, *F* is defined as $F\left(t\right)=\;F_{\lambda }\left(t\right)=\;c_{t_{d}}e^{-\;\text{λ}(t-t_{d})}$, where *t_d_* (hours) is the start of the decay period and $F\left(t_{d}\right)=c_{t}{}_{{}_{d}}$. Note that *F* approximates the average effect of cells stochastically switching to an inactive tolerant state on the total population dynamics.

Following similar reasoning, during a growth period, we define *F*(*t*) as $F_{\gamma }(t)=\;1-(1-q_{t_{g}})e^{-\;\text{γ}(t-t_{g})}$, where *t_g_* (hours) is the start of the growth period and *F*(*t_g_*) = $q_{t_{g}}$. Essentially, *F*_γ_(*t*) “undoes” the scaling from the decay period, restoring the population’s effective growth rate in characteristic time 1/γ (hours). Again, *F* represents an averaged perspective of a stochastic process, but in this case, it is that of cells switching from an inactive tolerant to active state. That is, we assume that cells lose their inactive state at some average rate γ (per hour) and can resume growth. Note that *F* is continuous and 0 ≤ *F*(*t*) ≤ 1 on (0, *T*_end_). The final form of *F* is determined by the temporally successive periods of either growth or decay conditions where continuity is maintained by specifying appropriate values of $c_{t_{d}}$ and $q_{t_{g}}$, respectively.

### (ii) Role of water availability.

As leaf wetness is generally difficult to accurately measure, Sentelhas et al. (29) conducted a field study showing that dew point is a good indicator of leaf wetness. A strong correlation was observed between the number of hours during which the DPD (the difference between the temperature and dew point) was <2°C and the number of leaf wetness hours on a cotton crop leaf canopy. This observation leads to the following key assumption: given the important role of free water in dictating bacterial cell dynamics on leaves, we assume that bacteria can grow (also depending on the temperature) on average if the DPD is <*T*_DPD_ (degrees Celsius); in contrast, the bacteria population will decay if the DPD is ≥*T*_DPD_ (degrees Celsius). Using results from Sentelhas et al. (29), we set the threshold *T*_DPD_ at 2°C. The following mathematical forms combine these ideas:

$$\text{μ}=\{\begin{array}{c} \;{\text{μ}}_{g}\left[y,\;T\left(t\right)\right]F_{\gamma }\left(t\right)\;\text{for}\;d\left(t\right)<T_{\text{DPD}}\\ \text{0}\;\text{for}\;d\left(t\right)\geq T_{\text{DPD}} \end{array}$$

and

$$k=\{\begin{array}{c} \;d\left(t\right)aF_{\lambda }\left(t\right)\;\text{for}\;d\left(t\right)\geq T_{\text{DPD}}\;\\ 0\;\text{for}\;d\left(t\right)<T_{\text{DPD}} \end{array}$$

where μ*_g_*[*y*, *T*(*t*)] (per hour) is the growth rate at time *t* as a function of the temperature *T*(*t*) (degrees Celsius), *d*(*t*) (degrees Celsius) is the DPD, a *T*_DPD_ of 2°C is the threshold below which the bacterial population experiences net growth and at or above which the bacterial population exhibits net decay, *a* (per hour-degree Celsius) is a scaling factor connected with the rate of decay induced by the DPD, and *F*_λ_(*t*) and *F*_γ_(*t*) are as defined above.

### (iii) Role of irradiance.

Irradiance in the context of this study broadly refers to the optical power received by leaf surfaces per unit area. Because radiometry is concerned with visible, infrared, and UV light, this model parameter represents the effect of light in plant growth chamber studies (mostly visible light) and in field trials (solar radiation). In order to quantify the effect of irradiance on pathogen decay, we consider the plant growth chamber study by Ottoson et al. (14), in which EcO157 population dynamics on lettuce were investigated under different temperature and light intensity conditions in a plant growth chamber. Using data from Fig. 3 in reference 14, we noticed that at a fixed temperature of 18°C and RH of 78%, hence at a fixed DPD of approximately 4°C, the decay rates of EcO157 increase as a function of light intensity, albeit at a decreasing rate of change with increased intensity. This provides evidence that the population decay rate is a concave function of irradiance, suggesting a saturation effect (Michaelis-Menten-type form). Based on these observations, we modify the decay function to be

$$k=\{\begin{array}{c} \;[1+\frac{L\left(t\right)}{b\;+\;L\left(t\right)}]\left[d\left(t\right)\right]aF_{\lambda }\left(t\right)\;\text{for}\;d\left(t\right)\geq T_{\text{DPD}}\;\\ 0\;\text{for}\;d\left(t\right)<T_{\text{DPD}} \end{array}$$

Where the term $\frac{L(t)}{b\;+\;L(t)}$ indicates the scaling effect of irradiance on decay; here, *L*(*t*) (in millimoles per square meter-second) is the irradiance, and *b* (in millimoles per square meter-second) is a positive constant representing the level of intensity at which the magnitude of the radiative effect on net decay is at 50%. It should be mentioned that the units (millimoles per square meter-second) for *L*(*t*) and *b* are relative to the experimental context of Ottoson et al. (14), but in field studies, *L*(*t*) and *b* can be expressed in watts per square meter or kilowatts per square meter, the typical units for measured solar radiation intensity.

### (iv) Growth as a function of temperature.

We applied the growth form from Ratkowsky et al. (17), namely, that $\sqrt{\;\text{μ}}=A\left(T\;-\;T_{0}\right)$, where *A* and *T*_0_ are constants; here, *T*_0_ corresponds to the minimal temperature at which growth can occur. Since $\sqrt{\;\text{μ}}=A\left(T\;-\;T_{0}\right)$, μ = α(*T* − *T*_0_)^2^, where α is expressed per hour-square degrees Celsius. Thus,

(1) $${\text{μ}}_{g}\left[y,T(t)\right]=\text{α}{\left[T\left(t\right)\;-\;T_{0}\right]}^{2}$$

if modeling exponential growth, especially when the growth period is short, and

(2) $${\text{μ}}_{g}\left[y,T(t)\right]=\text{α}{\left[T\left(t\right)-T_{0}\right]}^{2}\left(1-\frac{y}{K}\right)$$

if there is a determined carrying capacity (*K*) of >0 in the scenario of extended growth conditions, displaying logistic type growth.

### Final model.

Combining the above ideas, the finalized model is:

(3) $$\frac{\mathrm{dy}}{\mathrm{dt}}=\left(\mu -k\right)y,\;y\left(0\right)=y_{0}\;$$

where

(4) $$\text{μ}=\{\begin{array}{c} \;{\text{μ}}_{g}\left[y,T\left(t\right)\right]F_{\gamma }\left(t\right)\;\text{for}\;d\left(t\right)<T_{\text{DPD}}\\ 0\;\text{for}\;d\left(t\right)\geq T_{\text{DPD}} \end{array}$$

(5) $$F_{\gamma }(t)=\;1-(1\;-\;q_{t_{g}})e^{-\;\text{γ}(t-t_{g})}\;$$

and

(6) $$k=\{\begin{array}{c} \;[1\;+\frac{L\left(t\right)}{b\;+\;L\left(t\right)}]\left[d\left(t\right)\right]aF_{\lambda }\left(t\right)\;\text{for}\;d\left(t\right)\;\geq T_{\text{DPD}}\;\\ 0\;\text{for}\;d\left(t\right)\;<\;T_{\text{DPD}} \end{array}$$

(7) $$F_{\lambda }\left(t\right)=\;c_{t_{d}}e^{-\;\text{λ}(t\;-\;t_{d})}$$

where μ*_g_* [*y*, *T*(*t*)] (per hour) is the growth rate at time *t* and is expressed as either exponential growth (equation 1) or logistic growth (equation 2) depending on the context; *t_g_* (in hours) is the start of the growth period, *F*(*t_g_*) = $q_{t_{g}}$; γ (per hour) is the rate of switching from an inactive state; *d*(*t*) (degrees Celsius) is the DPD; *T*_DPD_ (degrees Celsius) is a threshold below which the bacterial population experiences net growth and at or above which the bacterial population exhibits net decay; *L*(*t*) (in millimoles per square meter-second, or watts per square meter or solar radiation) represents the intensity of light radiation; *b* (in millimoles per square meter-second, or watts per square meter for solar radiation) is a positive constant representing the intensity at which the magnitude of the radiative effect on net decay is at 50%; *a* (per hour-degree Celsius) is a scaling factor connected with the rate of decay induced by the DPD; *t_d_* (in hours) is the start of the decay period and *F*(*t_d_*) = $\;c_{t_{d}}$; and λ (per hour) is the rate of switching to an inactive state. Table 5 provides an organized, concise description of the variables and key parameters in the final model equations 3 to 7.

**TABLE 5:** Model variables and parameters

Symbol	Unit or value	Description
*y*	CFU/g leaf tissue	Total bacterial population
μ*_g_*	1/h	Bacterial growth rate
*k*	1/h	Bacterial death rate
*T*	°C	Ambient temp
λ	1/h	Rate of bacterial switching to inactive state
γ	1/h	Rate of bacterial switching from inactive state
*d*	°C	DPD
*T*_DPD_	2°C	DPD threshold
*L*	mmol/(m^2^·s) or W/m^2^	Intensity of light radiation or solar radiation
*b*	mmol/(m^2^·s) or W/m^2^	Level of intensity at which the magnitude of the radiative effect on net decay is at 50%
*a*	1/(h°·C)	Scale factor associated with the rate of decay induced by the DPD

### Parameter fitting.

Let *y_k_* (in CFU per gram of produce or just CFU, etc.) be the average bacterial population measurement (observed data) at time *t_k_* (in hours) and let $P_{k,\vec{\alpha }}$(CFU per gram of produce or just CFU, etc.) represent the corresponding model prediction (total population) at time *t_k_* relative to the parameter vector $\vec{\alpha }$. Since the population data typically span multiple orders of magnitude, we calculated the residuals as $e_{k,X\;}=\log_{10}y_{k}\;-\;\log_{10}P_{k,\vec{\alpha }}$. To determine the optimal model fit, we utilized the fminsearch function in MATLAB (MATLAB 2020b; The MathWorks, Inc., Natick, MA, USA) to determine the parameter vector $\vec{\alpha }$ that minimizes the 2-norm of the following function *E*:

(8) $$E{\left(\vec{\alpha }\right)}_{2}={\left(\underset{k}{\sum }\;e_{k,\vec{\alpha }\;}^{2}\right)}^{\frac{1}{2}}\;$$

Note that equation 8 computes the RMSE between observed data and model prediction relative to the parameter vector $\vec{\alpha }$. In addition to equation 8, we utilized a modified form to determine parameter values relative to two data sets simultaneously (see “Model fitting and prediction of E. coli population dynamics in plant chamber studies” in Results). To avoid parameter identifiability issues and thus maximize the information from the data (from Ottoson et al. [14]), the runs were fitted at two temperatures simultaneously, selecting the parameters *a* and λ, which minimized

(9) $$\hat{E}{\left(a,\text{λ}\right)}_{\;}\;={\left(\underset{k}{\sum }\;e_{k,11\;}^{2}\right)}^{\frac{1}{2}}+\;{\left(\underset{k}{\sum }\;e_{k,18\;}^{2}\right)}^{\frac{1}{2}}\;+\left|{\left(\underset{k}{\sum }\;e_{k,11\;}^{2}\right)}^{\frac{1}{2}}-\;{\left(\underset{k}{\sum }\;e_{k,18\;}^{2}\right)}^{\frac{1}{2}}\right|$$

i.e., incorporating the sum of the error as well as the difference of the error from the runs for 11°C and 18°C, respectively.
