# Supplementary material for: Predictive Population Dynamics of Escherichia coli O157:H7 and Salmonella enterica on Plants: a Mechanistic Mathematical Model Based on Weather Parameters and Bacterial State
Source: Appl Environ Microbiol. 2023 Jun 22;89(7):e00700-23. doi: 10.1128/aem.00700-23 (PMC10370311; doi:10.1128/aem.00700-23)
Supplement: Supplemental file 1 — Supplemental material. Download aem.00700-23-s0001.pdf, PDF file, 0.1 MB [file aem.00700-23-s0001.pdf]

Supplemental Text

Value of the parameter *b* for field application:

In section 1 of the Results, we determined b = 70 mmol/(m^2s), in the case of exposing EcO157 on lettuce to light intensity of 400 mmol/(m^2s) in a controlled plant chamber. Using the assumption that

$$\frac{b_{lab}}{Average\ Intenstity_{lab}} \approx \frac{b_{field}}{Average\ solar\ radiation_{field}},$$

we determined $b_{field} \approx$ 100 W/m^2. Thus, for the field application in subsections 4.1 and 4.2 of the Results, we set b = 100 W/m^2.

Calculation of Dew point from Temperature and RH:

Following Lawrence 2005 (1) , we used the expression for the dew point temperature t_d (°C) as

$$t_d = \frac{B_1\left[\ln\left(\frac{RH}{100}\right) + \frac{A_1 t}{B_1 + t}\right]}{A_1 - \ln\left(\frac{RH}{100}\right) - \frac{A_1 t}{B_1 + t}}$$

where RH is the relative humidity, t is the temperature (°C), A_1 = 17.625, and B_1 = 243.04 (°C).

Calculations for growth rates under lab-controlled conditions:

In section 2 of results, for the model application for the dynamic high RH regime, we used μ_g = 0.027 (1/h) for the growth rate. This value comes from calculations that adapted information on

the minimal temperature for *Salmonella* growth from Table 1 of Koseki and Isobe 2005 (2) and the average temperature for the high (dynamic) regime experiments in López-Gálvez et al. 2018 (3).

In section 3 of the results, in the context of applying the model to the "dry-wet" experiment from Brandl 2006 (4), the growth dynamic, from 96 to 168 h post-inoculation, was modeled using $\mu_g = 0.2621\left(1 - \frac{y}{K}\right)$. Here 0.2621 quantifies the growth rate at Temp = 26ºC (determined from data from Figure 1 and 5A in Brandl and Mandrell 2002 (5)) and K = 6 $\log_{10}$ CFU/g (determined from the maximum population value measured during the "dry-wet" experiment in Brandl 2006 (4)) .

References

1. Lawrence MG. 2005. The relationship between relative humidity and the dewpoint temperature in moist air: A simple conversion and applications. Bull Amer Meteorol Soc 86:225-234.
2. Koseki S, Isobe S. 2005. Prediction of pathogen growth on iceberg lettuce under real temperature history during distribution from farm to table. Int J Food Microbiol 104:239-248.
3. López-Gálvez F, Gil MI, Allende A. 2018. Impact of relative humidity, inoculum carrier and size, and native microbiota on *Salmonella* ser. Typhimurium survival in baby lettuce. Food Microbiol 70:155-161.
4. Brandl MT. 2006. Human pathogens and the health threat of the phyllosphere, p 269-285. *In* Bailey MJ, Lilley AK, Timms-Wilson TM, Spencer-Philips PTN (ed), Microbial ecology of aerial plant surfaces CABI Publishing, Wallingford, United Kingdom.
5. Brandl MT, Mandrell RE. 2002. Fitness of *Salmonella enterica* serovar Thompson in the cilantro phyllosphere. Appl Environ Microbiol 68:3614-3621.
